# History of the Royal Academy of Sciences for the Year 1780; with the Mathematical and Philosophical Memoirs for That Year, Taken from the Registers of the Academy

**Published:** 1785

**Authors:** 


II. *Histoire de l'Academie Royale des Sciences. Année* 1780. *Avec les Memoires de Mathematique et de Physique, pour la meme Année, tirés des Registres de cette Academie.* i. e. *History of the Royal Academy of Sciences for the Year* 1780; *with the mathematical and philosophical Memoirs for that Year, taken from the Registers of the Academy.* 4to. Paris, 1784. 756 pages.

IN the historical part of this volume we meet with the eulogies of the late Messrs. Lieutaud and Bucquet. These we shall pass over, as we have already had occasion to mention the eulogies of these two physicians, published in the Memoirs of the Royal Medical Society*.

Among the memoirs, which form the second part of the volume, are the following:

1. *On the Combination of Oils with Earths, volatile Alkali, and metallic Substances. By M.* Bertholet.—The idea of these experiments was suggested, it seems, by the affinity which soap has in its properties to the neutral salts. This resemblance led our author to suppose that oil, like the acids, might be susceptible of a great number of combinations, which had hitherto been overlooked. M. Costel, he observes, was the first writer who described † the method of combining oil with calcareous earth, by pouring a solution of soap into lime water; the lime in this case uniting with the oil of the soap, and forming with it an insoluble substance, leaving the caustic alkali at liberty. M. Thouvenel, who has mentioned this circumstance in his *Analyse des Eaux de Contrexeville*, recommends it to the attention of physicians, who frequently prescribe soap and lime water, and probably without being aware of the decomposition that

* See the 5th vol. of this work, p. 116 and 117.

† Analyse des Eaux de Pougues.

takes takes place, and renders the cauſtic alkali the active part of the remedy.

By mixing a ſolution of common ſoap with a ſolution of ſal ammoniac, our author inſtantly procured what he calls an *ammoniacal ſoap*, which is formed by the union of the fixed alkali of the ſoap with the acid of the marine ſalt, while the volatile alkali unites with the oil. He recommends this ſoap as preferable to common ſoap for medicinal purpoſes; and it has theſe advantages, he obſerves, over Starkey's ſoap, that it may be eaſily and ſpeedily prepared, is always the ſame, and keeps well in cloſe veſſels. He is aware that a mixture of volatile alkali and oil has long been employed in phyſic, and that a compoſition of this kind is directed in the *Pharm. Londin.* under the name of *Linimentum volatile*; but this, he obſerves, is entirely different from the ammoniacal ſoap he has deſcribed.

In the courſe of the paper, the author gives the reſults of his experiments in combining oils with earths and metallic ſubſtances. The combination procured by mixing a ſolution of common ſoap with a ſolution of corroſive ſublimate, is, he thinks, likely to be uſeful in phyſic.

11. *Ob-*

11. *Obſervations on the phoſphoric Acid of Urine. By* the Same.— It has lately been aſſerted by a very ingenious chymiſt, that no phoſphoric acid exiſts originally either in the urine or the bones, but that it is formed by the putrefaction and other means employed for procuring it. M. Bertholet combats this opinion with a very ſimple experiment, which conſiſts in pouring lime water into freſh urine. By this mixture, a precipitate is formed, which he finds to be a phoſphoric ſalt with a calcareous baſis. Five parts of this ſalt, we are told, contain three of phoſphoric acid. Theſe proportions, he obſerves, may differ from circumſtances of age, ſex, diſeaſe, climate, &c.; but as urine, from its containing this acid, reddens paper coloured with tincture of Tourneſol, he thinks we may, with a little attention, be able to aſcertain, by the degree of redneſs the paper acquires, the proportion of acid contained in the urine.

M. Bertholet has already convinced himſelf, that the urine of different perſons is very different in this reſpect, independently of its colour and other ſenſible qualities. For example, the urine of a gouty patient, which he examined for ſome time, conſtantly yielded only about a third third of the precipitate he obtained from his own urine; but during two fits of the gout, the urine of this perſon was found to yield nearly the ſame proportion of precipitate as his own did.

M. Bertholet has obſerved likewiſe, that the ſweat gives a red colour to paper dipped in tincture of Tourneſol, but he has ſeen no ſuch effect produced by the ſaliva.

III. *Analyſis of a new ſpecies of Biſmuth. By* M. Sage.

IV. *Method of rendering white and tranſparent, Phoſphorus that is opaque, and of a yellow or red Colour. By* the Same. — This method of rendering Phoſphorus tranſparent, conſiſts in melting it in a ſand bath, when the opaque part, which colours it, floats on the ſurface, and may be eaſily ſeparated. By this proceſs, the Phoſphorus is rendered more pure, the opaque part being but ſlightly luminous. In this paper alſo M. Sage ſpeaks of the ſolution of phoſphorus in ſpirit of wine, of which, it ſeems, twelve hundred parts are requiſite to diſſolve one of phoſphorus. This ſolution is not luminous, unleſs water is added to it; and then the phoſphorus is ſeparated, and floating on the ſurface, yields a faint light. If the ſolution is ſet on fire, it emits not not a blue flame, like ſpirit of wine, but a green flame like phoſphorus, when burnt ſlowly.

v. *Remarks on a new Species of yellow martial Precipitate.* *By* the Same.

vi. *New Obſervations on Sulphur.* *By* M. Fougeroux de Bondaroy.

vii. *Eſſay on the Method of purifying the Air of Ships.* *By* M. de Bory. — The method here recommended is that of our countryman Sutton, with ſome variations, adapted to the particular ſituation of the kitchen in French ſhips of war.

viii. *Inquiries concerning the Natnre of animal Subſtances, and their affinity to vegetable Subſtances.* *By* M. Bertholet, — We here learn, that if nitrous acid be poured on ſilk, wool, hair, or ſkin, and diſtilled, a certain quantity of animal oil, different from that which forms the fat, is obtained, and likewiſe a portion more or leſs conſiderable of an acid, reſembling acid of ſugar. This acid, therefore, is common both to vegetable and animal ſubſtances; but the oil found in the latter, our author obſerves, ſeems to be peculiar to them.

[ *To be continued.* ]

III. *Me-*

